# Evolution of All-or-None Strategies in Repeated Public Goods Dilemmas

**DOI:** 10.1371/journal.pcbi.1003945

**Published:** 2014-11-13

**Authors:** Flávio L. Pinheiro, Vítor V. Vasconcelos, Francisco C. Santos, Jorge M. Pacheco

**Affiliations:** 1 Centro de Biologia Molecular e Ambiental da Universidade do Minho, Braga, Portugal; 2 INESC-ID & Instituto Superior Técnico, Universidade de Lisboa, Taguspark, Porto Salvo, Portugal; 3 Centro de Física da Universidade do Minho, Braga, Portugal; 4 ATP-group, CMAF, Instituto para a Investigação Interdisciplinar, Lisboa, Portugal; 5 Departamento de Matemática e Aplicações da Universidade do Minho, Braga, Portugal; Brain and Spine Institute (ICM), France

## Abstract

Many problems of cooperation involve repeated interactions among the same groups of individuals. When collective action is at stake, groups often engage in *Public Goods Games* (**PGG**), where individuals contribute (or not) to a common pool, subsequently sharing the resources. Such scenarios of repeated group interactions materialize situations in which direct reciprocation to groups may be at work. Here we study direct group reciprocity considering the complete set of reactive strategies, where individuals behave conditionally on what they observed in the previous round. We study both analytically and by computer simulations the evolutionary dynamics encompassing this extensive strategy space, witnessing the emergence of a surprisingly simple strategy that we call *All-Or-None* (**AoN**). **AoN** consists in cooperating only after a round of unanimous group behavior (cooperation or defection), and proves robust in the presence of errors, thus fostering cooperation in a wide range of group sizes. The principles encapsulated in this strategy share a level of complexity reminiscent of that found already in 2-person games under direct and indirect reciprocity, reducing, in fact, to the well-known *Win-Stay-Lose-Shift* strategy in the limit of the repeated 2-person *Prisoner's Dilemma*.

## Author Summary

The problem of cooperation has been a target of many studies, and some of the most complex dilemmas arise when we deal with groups repeatedly interacting by means of a *Public Goods Game* (**PGG**), where individuals may contribute to a common pool, subsequently sharing the resources. Here we study generalized direct group reciprocity by incorporating the complete set of reactive strategies, where action is dictated by what happened in the previous round. We compute the pervasiveness in time of each possible reactive strategy, and find a ubiquitous strategy profile that prevails throughout evolution, independently of group size and specific **PGG** parameters, proving also robust in the presence of errors. This strategy, that we call *All-Or-None* (**AoN**), consists in cooperating only after a round of unanimous group behavior (cooperation or defection); not only is it conceptually very simple, it also ensures that cooperation can self-sustain in a population. **AoN** contains core principles found, e.g., in the repeated 2-person Prisoner's Dilemma, in which case it reduces to the famous *Win-Stay-Lose-Shift* strategy.

## Introduction

The emergence and sustainability of cooperation constitutes one of the most important problems in social and biological sciences [1]. It revolves around the clash between individual and collective interest, which becomes particularly clear when one considers the evolution of collective action involving *Public Goods Games* (**PGG**), such as the stereotypical *N-person Prisoner's Dilemma* (**NPD**) [2], [3]. In the absence of additional mechanisms, such as the presence of *thresholds* [4], [5], *risk* [6], an *embedding network of interactions* [7]–[12], *institutions* [13]–[15], *punishment* or *voluntary participation* [16]–[19], evolutionary game theory predicts a population fated to fall into a tragedy of the commons [20].

Collective action problems, however, often involve repeated interactions between members of the same group [21]–[23], as exemplified by the repeated attempts from country leaders to cooperate in reducing emissions of greenhouse gases [6], [24]–[29] or in finding a solution to the Euro monetary crisis [30]–[32]. In such scenarios, where collective action is more difficult to achieve in larger groups [6], one is naturally led to question whether a generalization of the direct reciprocity [33] mechanism to problems of collective action may provide an escape hatch to the aforementioned tragedy of the commons. Moreover, *N*-player interactions pose many additional difficulties, in particular in what concerns the emergence of reciprocation: If one interacts repeatedly in a group of N-players it is hard to identify towards whom should one reciprocate [3]. In fact, only recently direct reciprocity has been generalized to **PGG**s [22], [23], studying the co-evolution of unconditional defectors with generalized reciprocators, that is, individuals who, in a group of size *N*, only cooperate if there were at least *M* (0≤*M*≤*N*) individuals who cooperated in the previous round. Results show [22], [23] that generalized reciprocators are very successful in promoting cooperation. Moreover, for a given group size *N*, there is a critical threshold level of fairness, M^*^, at which reciprocation optimizes the emergence of cooperation [22].

Generalized reciprocators [22] provide an intuitive generalization of the **TFT** strategy to repeated *N*-player games. However, and despite the underlying intuition, they constitute but a small subset of all possible individual (reactive) strategies one can envisage in a group of size *N*.

Here we explore the complete set of reactive strategies that individuals may adopt when engaging in repeated *Public Goods Games* with *N*-1 other individuals, assuming that the decision to cooperate or not is based on the behavioral decisions of the group in the previous round (see below). We find that, in the context of *Public Goods Games*, a reactive strategy not belonging to the set of generalized reciprocators emerges as ubiquitous, ensuring the emergence and sustainability of cooperation.

## Models

Let us consider a finite and *well-mixed* population of *Z* individuals, who assemble in groups of size *N* randomly formed, and play a repeated version of the **NPD** [34]. In each round individuals either *cooperate* (**C**) by contributing an amount *c* to a public good or *defect* (**D**) by not doing so. The aggregated contributions of the group are multiplied by an enhancement factor *F* and equally divided among the *N* individuals of the group. Hence, in each round, **D**s achieve a payoff of 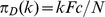, while **C**s attain 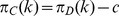 where *k* is the number of contributions in that round. We consider a repeated **PGG** with an undetermined number of rounds, such that at the end of each round, another round will take place with probability *w* [3], leading to an average number of rounds — *m* — given by *m* = (1−*w*)^−1^. At the beginning of each round (with the exception of the first), each individual decides to contribute (*i.e.* to play ***C***) or not (*i.e.* to play ***D***), depending on the total number of contributions that took place in the previous round.

Each strategy *S_i_* defines how an individual behaves in each round (*i.e.* if she/he decides to cooperate or defect) and is encoded in a string with *N*+2 bits (*b^−1^b^0^b^1^…b^N−1^b^N^*). The first bit (*b^−1^*) dictates the behavior in the initial round, while the remaining *N*+1 bits (*b^0^b^1^…b^N−1^b^N^*) correspond in sequence to the player's behavior depending on the number of ***C***s in the previous round. In this definition a bit 1 corresponds to a *cooperative* act and a bit 0 to a *defective* one. Hence, one obtains a maximum of 2*^N^* ^+2^ strategies, corresponding to all possible combinations of 0 s and 1 s in a string of size *N*+2.

We consider groups of *N* individuals, randomly sampled from a finite population of size *Z*, playing a repeated **NPD**. Individuals revise their strategies through the Fermi update rule [35]–[38], a stochastic birth-death process with mutations. At each time step a randomly selected individual *A* (with strategy *S_A_* and fitness 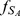) may adopt a different strategy *i*) by mutation with probability *μ* or *ii*) by imitating a random member *B* of the population (with strategy *S_B_* and fitness 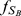) with probability 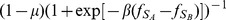, where *β* is the *intensity of selection* that regulates the randomness of the decision process. The fitness of each strategy 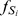 is the average payoff attained over all rounds and possible groups by individuals adopting strategy *S_i_*. It is well known that execution errors profoundly affect the evolutionary dynamics of repeated 2-person games [39]–[45]. Consequently, we shall also consider that, in each round, and after deciding to contribute or not according to *b^q^*, an individual may act with the opposite behavior (1−*b^q^*) with a probability *ε*, thus making an execution error.

## Results/Discussion

Let us start by investigating the evolutionary dynamics of the population in the *small mutation limit* approximation [46]. This allows us to compute analytically the relative pervasiveness of each strategy in time. It is noteworthy, however, that the results obtained through this approximation remain valid for a wide range of mutation probabilities, as we show explicitly in the Supporting Information (**SI**) via comparison with results from computer simulations. In a nutshell, and whenever mutations are rare, a new mutant that appears in the population will either get extinct or invade the entire population before the occurrence of the next mutation. Hence, in each time-step there will be, at most, 2 strategies present in the population, which allows one to describe the evolutionary dynamics of the population in terms of an embedded (and reduced) Markov Chain with a size equal to the number of strategies available. Each state represents a monomorphic population adopting a given strategy, whereas transitions are defined by the fixation probabilities of a single mutant [47]. The resulting stationary distribution *τ_i_* will then indicate the fraction of time the population spends in each of the 2*^N^* ^+2^ states (or strategies *S*_i_). We shall also make use of *τ_i_* to compute the fraction of time the population spends in a configuration/strategy with *b_i_^q^* = 1, a quantity we call *stationary bit strategy*, defined as 
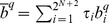
, where 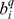 corresponds to the bit *q* of strategy *i*. The *stationary bit strategy* allows us to easily quantify the relative dominance of each behavior and extract the most pervasive strategic profiles.

**Figure 1:**
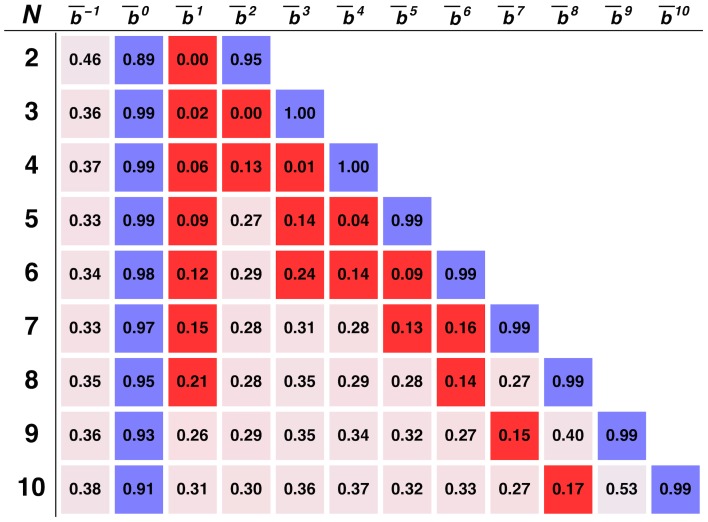
Stationary bit distribution as a function of *N*. Each bit (square) corresponds to the weighted sum of the fraction of time (*i.e.* the analytically computed stationary distribution) the population spends in strategy configurations in which *b^q^* = 1. Blue (red) cells identify those bits that are employed at least ¾ of the time with value *b^q^* = 1.0 (*b^q^* = 0.0). The analysis provided extends for groups sizes (*N*) between 2 and 10 (rows). Other model parameters: *Z* = 100, *β* = 1.0, *F/N* = 0.85, *w* = 0.96, *ε* = 0.05, *μ*≪1/*Z*.

Figure 1 shows the *stationary bit distribution*, 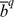, for different group sizes. Colored cells highlight those bits (*b^q^*) that retain the same value more than 75% of the time, with 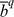
≥0.75 (blue) and 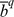
≤0.25 (red). For simplicity, we associate this feature with what we call *dominant bit*.

Analysis of the *stationary bit distributions* for different group sizes under small error probabilities puts into evidence the overall evolutionary success of strategies that conform with a particular profile: *b^0^* = *b^N^* = 1 and *b^q^* = 0 for 0<*q*<*N*. A similar trend is obtained if instead we analyze the stationary distribution *τ*_i_ for all possible strategies *S_i_*: This strategy — or minor variations on this profile (see below) — shows the highest prevalence for a wide range of parameters even in the absence of errors of execution (see **SI**). The philosophy encapsulated in this strategy is a simple yet efficient one: cooperating only after a round of unanimous group behavior (cooperation or defection). Hence we refer to this strategy as *All-Or-None* (**AoN**), highlighting the two situations in which these individuals are prone to cooperate. As group size increases, so does the number of expected errors per round, which leads to an overall reduction of the number of *dominant bits* found in the intermediate sector (*i.e. b^q^* for 0<*q*<*N*) without affecting the “edge bits”, which again reveals the prevalence of **AoN** behaviour in the population.

A monomorphic population of **AoN** players can easily sustain unanimous group cooperation, even in the presence of errors. Indeed, after an occasional individual defection, a round of full defection ensues, resuming back to unanimous cooperation in the following round. Therefore, **AoN** allows a prompt recovery from errors of execution, which constitutes a key feature that allows cooperation to thrive.

**Figure 2:**
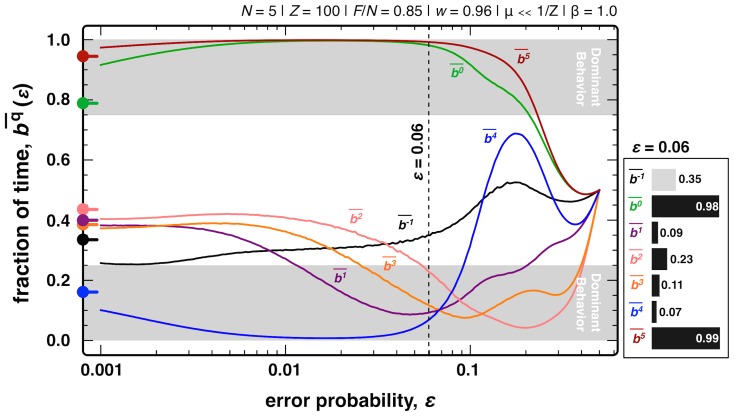
Stationary bit distribution as a function of the error rate. We plot (log-linear scale) the fraction of time the population spends in a strategy with *b^q^* = 1 for a broad range of error probabilities *ε*. Circles on the left indicate the values obtained for *ε* = 0.0, gray areas show the range of values for which bits were defined to have a dominant behavior. Note that for *ε* = 0.5 all strategies behave randomly. The bar plot on the right shows the results for *ε* = 0.06 (vertical dashed line). Other model parameters: *Z* = 100, *β* = 1.0, *N* = 5, *F/N* = 0.85, *w* = 0.96 and *μ*≪1/*Z*.

To investigate the robustness of **AoN** we show, in Figure 2, the effect of execution errors on the *stationary bit distribution* (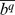) for a fixed group size (here *N* = 5): Clearly, both *b^0^* and *b^N^* remain associated with cooperation for a wide range of error probabilities (*ε*≤0.2). The internal bits, in turn, remain qualitatively close to the **AoN** profile (*i.e. b^q^* = 0 for 0<*q*<*N*), undergoing changes as the error rate increases, allowing an efficient resume into full cooperation, after (at least) one behavioral error. In particular, for 0.01<*ε*<0.1, evolution selects for defection in bits *b^1^* to *b^N−1^*, with particular incidence to adjacent bits of *b^0^* and *b^N^*, allowing a fast error recovery. This feature gets enhanced with increasing *ε*. For larger values of *ε* (*ε*>0.1), unanimity becomes less likely and we witness an adaptation of the predominant strategy that acts to reduce the interval of bits in which defection prevails. In other words, it is as if execution errors redefine the notion of unanimity itself or, alternatively, individuals become more tolerant as execution errors become more likely. It is also noteworthy that the non-monotonous response to errors shown in Figure 2 has been previously observed in other evolutionary models of cooperation [48] where intermediate degrees of stochasticity emerge as maximizers of cooperation. We confirmed that the results remain qualitatively equivalent for different group sizes.

**Figure 3:**
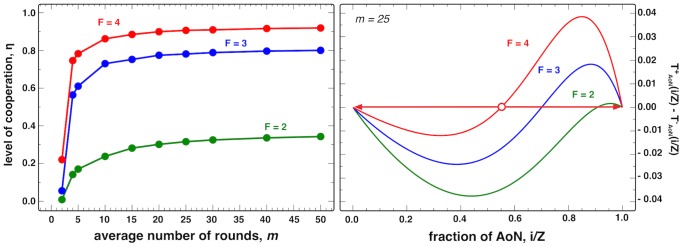
Left: Level of cooperation as a function of average number of rounds. *m* for three different values of the enhancement value *F* (4, 3 and 2) with *N* = 5 and in the absence of behavioral errors. **Right:** Gradients of Selection [5] for the evolutionary game between **ALLD** and **AoN** (*b^−1^* = 0, *N* = 5, w = 0.96 or *m* = 25; other model parameters: *Z* = 100 and *β* = 1.0).

In the following we investigate the relevant issue of asserting whether the introduction of this strategy can efficiently promote the average fraction of cooperative actions. The level of cooperation, *η*, may be defined as the average number of contributions per round divided by the maximum number of contributions possible. Denoting by *C_i_* the average number of contributions per round associated with strategy *S_i_*, *η* reads 
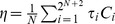
, where *τ_i_* is the fraction of time the population spends in the configuration *S_i_* and *N* is the group size. As shown in Figure 3, the overall levels of cooperation remain high as long as the average number of rounds is sizeable (left panel, for different values of the **PGG** enhancement factor *F*).

The success of **AoN** can also be inferred by assessing its evolutionary chances when interacting with unconditional defectors (**AllD**). To do so, we compute the gradient of selection [5] — *G*(*k*) — which provide information on the most likely direction of change of the population configuration with time. This is given by the difference between the probabilities of increasing and decreasing the number of **AoN** players in a population of *k* **AoN**s and *Z-k* **AllD**s. The result is depicted in the right panel of Figure 3, a profile characteristic of a coordination game, in which case the **AoN** strategy dominates whenever the population accumulates a critical fraction of **AoN** players. Moreover, the size of coordination barrier decreases with increasing values of the enhancement factor *F*. In the **SI** we further show that the location of the coordination point is rather insensitive to other game parameters, in particular when the number of rounds is large. Notably, the evolutionary chances of the **AoN** strategy remain qualitatively independent from alterations on the first bit (*b^−1^*). Similarly, we have checked the robustness of the **AoN** strategy when interacting with random strategists (**RS**), i.e., individuals that cooperate or defect with equal probability. It can be shown that both **AoN** and **AllD** are advantageous with respect to **RS** strategists (regardless of their prevalence in the population), while these should drive **AllC** to extinction. Finally, contrary to the generalized versions of **TFT** strategies, in the presence of errors, the **AoN** strategy is robust to invasion of unconditional cooperators (**AllC**) by random drift, as the former can efficiently exploit the latter.

To sum up, we have shown that the strategy **AoN** emerges as the most viable strategy that leads to the emergence of cooperation under repeated **PGG**s. This strategy, despite its remarkable simplicity, cannot be encoded within the subspace of generalized reciprocators studied before in this context [22]. When we consider individuals capable of making behavioral errors, **AoN** is dominant as suggested by analyzing both the *stationary bit strategy* (Figures 1 and 2) and the stationary distribution in the monomorphic configuration space (**SI**). More importantly, our results suggest that **AoN** dominates independently of the group size and over a wide range of error rates.

Previous works have identified similar strategy principles in different contexts. For instance, the *Win-Stay-Lose-Shift* [39]–[41], [49] strategy discovered in the context of the repeated 2-person Prisoner's Dilemma constitutes the *N* = 2 limit of **AoN**. In the context of repeated N-Person games on the multiverse [34], the strategy entitled *generic Pavlov* [50] encapsulates a behavioral principle which is similar to that underlying **AoN**. In fact, one may argue that the principles underlying **AoN** may very well be ubiquitous: The simplicity of this strategy can be seen as equivalent — in the context of problems of collective action [5], [6], [14] involving *Public Goods Games* — to the simplicity of *tit-for-tat* or *Win-Stay-Lose-Shift* strategies discovered in the context of 2-person direct reciprocity, or the *stern-judging* social norm of indirect reciprocity [51]. In these cases, we observe a fine balance between strict replies towards defective actions and prompt forgiving moves, allowing the emergence of unambiguous decision rules (or norms) that may efficiently recover from past mistakes. Thus, despite the inherent complexity of N-person interactions and the individual capacity to develop complex strategies, it is remarkable how evolution still selects simple key principles that lead to widespread cooperative behaviors.

## Supporting Information

Text S1 **Supporting text.** (containing 4 additional figures) provides additional details concerning the methodology adopted and investigates the impact of *i*) mutation rates and *ii*) the evolution in the absence of execution error rates in the evolutionary dynamics of the *N-Person* repeated *Prisoner's Dilemma*.
(RAR)
